# Laser-Induced Au Catalyst Generation for Tailored ZnO Nanostructure Growth

**DOI:** 10.3390/nano13071258

**Published:** 2023-04-02

**Authors:** Sebastien Durbach, Lars Schniedermeyer, Anna Marx, Norbert Hampp

**Affiliations:** Department of Chemistry, University of Marburg, Hans-Meerwein Str. 4, 35032 Marburg, Germany

**Keywords:** ZnO-nanostructures, gradual ZnO growth manipulation, spatial-selective ZnO growth, laser-induced catalyst generation, thermal chemical vapor deposition

## Abstract

ZnO nanostructures, semiconductors with attractive optical properties, are typically grown by thermal chemical vapor deposition for optimal growth control. Their growth is well investigated, but commonly results in the entire substrate being covered with identical ZnO nanostructures. At best a limited, binary growth control is achieved with masks or lithographic processes. We demonstrate nanosecond laser-induced Au catalyst generation on Si(100) wafers, resulting in controlled ZnO nanostructure growth. Scanning electron and atomic force microscopy measurements reveal the laser pulse’s influence on the substrate’s and catalyst’s properties, e.g., nanoparticle size and distribution. The laser-induced formation of a thin SiO_2_-layer on the catalysts plays a key role in the subsequent ZnO growth mechanism. By tuning the irradiation parameters, the width, density, and morphology of ZnO nanostructures, i.e., nanorods, nanowires, and nanobelts, were controlled. Our method allows for maskless ZnO nanostructure designs locally controlled on Si-wafers.

## 1. Introduction

Regarding the ever-growing demand for new functional materials, semiconductor structures in the micro- and nanoscale remain of high interest in the research community. Due to their physical properties e.g., wide band gap [1] (~3.4 eV) and large exciton binding energy [2] of 60 meV, ZnO nanostructures are promising candidates for future electronic [3] or optoelectronic [4] devices as in UV-lasers [5], solar cells [6] or sensoric [7,8] applications. The generation of diverse ZnO nano-morphologies may be achieved by both wet [9,10] and dry [11,12,13] methods. In general dry approaches, e.g., thermal chemical vapor deposition (t-CVD), are more abundant and controllable. For ZnO nanostructure growth the vapor-solid- (VS) and the vapor-liquid-solid- (VLS) reactions are of relevance [11]. Control of which mechanism is dominating the growth process and hence guides the morphology expression is commonly controllable by adjusting the process variables, e.g., temperature [8,14] and reaction gas composition [15,16]. Most commonly a noble metal layer, e.g., gold, is applied before the growth process. Heating the thin film leads to thermal dewetting and nanoparticle formation. Upon introducing a semiconductor vapor source, e.g., through a carbothermal reaction [17], the nanoparticles act as catalysts (VLS) or nucleation points (VS) inducing the ZnO nanostructure growth. Spatial-selective control of the semiconductor growth is essential for the design of functional devices. The catalyst may be patterned by using a mask [18], by direct laser writing [19], or by lithographic methods [20], leading to binary growth regulation. Nanostructure growth is either fully suppressed or enabled.

In this work, we first analyze the ZnO morphology occurrence in dependence on underlying growth parameters and thus mechanisms. This allows the differentiation between ZnO modifications mediated by the growth parameters and the pulsed laser-induced dewetting (PLiD) of the catalyst layer (Au thin film). Particle analysis was conducted by electron microscopy images to investigate the type, size, and density of laser-generated catalysts. The growth of ZnO nanostructures can consequently be adjusted by the spatial-selective laser-induced catalyst generation, leading to the manifestation of various ZnO morphologies and sizes on a single substrate. Finally, the laser-induced formation of a passivating SiO_2_ layer and its effect on the ZnO growth was investigated.

## 2. Materials and Methods

**Figure 1:**
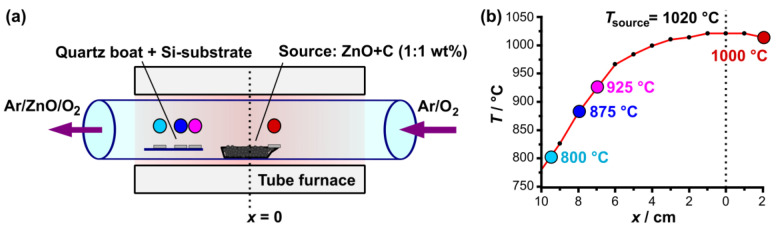
Schematic representation of the tube furnace system (**a**) and the corresponding temperature profile during the ZnO growth (**b**). The sample positions are mapped to their respective process temperatures by the colored indicators. The source is located at *x* = 0 cm and consists of a 1:1 mixture (weight) of ZnO and graphite powder.

The silicon samples used in this study were mechano-chemically polished boron-doped (100) (Siegert Wafer, Aachen, Germany) single crystals with a native oxide layer of ca. 1.5 nm. The wafer fragments (ca. 1 × 1 cm) were coated with gold (0.6–5.4 nm) using a 108auto vacuum coater system (Cressington, Watford, UK). The thickness of thin films was measured with single-wavelength (633 nm) ellipsometry. ZnO growth was performed in a horizontal tube furnace inside of a quartz glass tube with an inner diameter of 40 mm and a length of 650 mm. The source material consisted of a mixture of ZnO (abcr, 99.99%) and graphite powder (99.9%) with a weight ratio of 1:1. The mixture (~1 g) was placed in an alumina boat at the center of the tube (Figure 1, *x* = 0 cm). The wafer fragments were placed on a quartz glass slab at a distance of 7 cm, 8 cm, and 9.5 cm downstream and at 2 cm upstream (edge of alumina boat). The sample placement and furnace setup are depicted in Figure 1a. Figure 1b shows the temperature gradient inside the tube furnace. The sample placements are mapped to their respective temperatures with colored indicators. The setup’s heating curve is displayed in the supporting information (SI, Figure S1). The source was heated to a temperature of 1020 °C, initiating the generation of Zn-vapor according to the carbothermal reaction (1) and/or (2):

ZnO_(s)_ + C_(s)_ → Zn_(g)_ + CO_(g)_ (1)

ZnO_(s)_ + CO_(g)_ → Zn_(g)_ + CO_2(g)_ (2)

Before each growth process, the tube was evacuated to a pressure of 2 mbar for several minutes. During the growth process, the pressure was kept at 10–15 mbar. As carrier gas pure argon (Nippon gases, Madrid, Spain, 99.999%, 1–1.5 bar) was optionally mixed with a mixture of argon and oxygen (Sanarc, 8% O_2_). All experiments were conducted with a steady Ar (+O_2_) gas flow of 30 sccm. Note that minimal leakage in the system has to be assumed, allowing the entry of O_2_ in the ppm range, needed to initiate ZnO formation [16]. The quartz glass tube was cleaned with concentrated hydrochloric acid (HCl, 37%) before every growth process to ensure uniform growth parameters. This prevents a shift in the ratio of VS to VLS growth mediated by a variation of the carbon concentration [11].

Laser irradiation was performed with a frequency-doubled Nd:YVO_4_ diode-pumped solid-state (DPSS) laser (explorer XP 532-5, Newport Corporation, Irvine, CA, USA) emitting ≈ 8 ns pulses (FWHM) of *λ*_0_ = 532 nm wavelength in TEM_00_ (M^2^ < 1.1) mode. The nanosecond-laser pulses were focused to a spot radius of 50 μm (1/e^2^) at normal incidence to the substrate surface. The spot size was measured by fitting data from the knife-edge method at multiple z-heights. The beam was scanned over the substrate by a galvanometer scan head (SCANgine 14-532, Scanlab, Puchheim, Germany). More details on the laser setup used for irradiation are found in the SI (Figure S2). The laser power was measured with an LM-80V detector head (FieldMax II, Coherent, Santa Clara, CA, USA). The polarization state of the laser was measured with a polarimeter (Thorlabs TXP 5004 with the PAN5710VIS scan head). Scanning electron microscopy (SEM) was performed on a CrossBeam-Workstation (Gemini 2, Carl Zeiss, Oberkochen, Germany) with a silicon-drift EDX-detector (Ultim^®^Max. Oxford Instruments, Abingdon, UK). Cross-sections of the samples were achieved by splitting the substrates after ZnO growth. Atomic force microscopy (AFM, Nanoscope IV, Bruker, Billerica, MA, USA) was conducted in contact mode using sharp nitride layer (SNL) tips (*f* = 56 kHz, *k* = 0.24 N/m). Gwyddion, an open-source software, was used for the analysis of the particle’s size and density, as well as for AFM image processing.

## 3. Results and Discussion

### 3.1. ZnO Growth Mechanism and Morphologies

**Figure 2:**
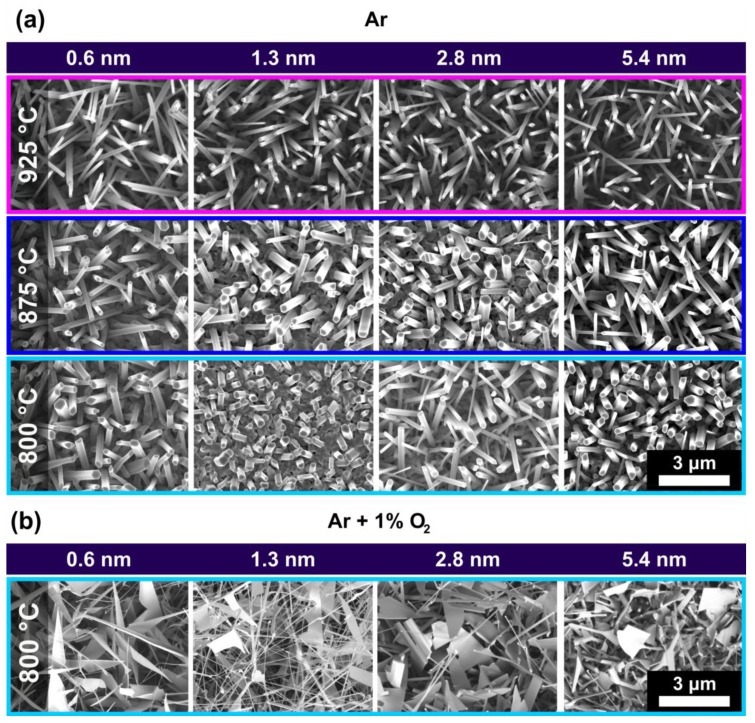
(**a**) Typical ZnO-nanostructures generated in a pure Ar–atmosphere at different temperatures and gold thin film layer thicknesses (0.6–5.4 nm) (*p* = 12 mbar, *t* = 10 min, *T_source_* = 1020 °C). (**b**) Typical ZnO-nanostructures generated under the same conditions as (**a**) but in an Ar + O_2_(1%)–atmosphere. The colors match the temperatures and sample positions illustrated in Figure 1.

As the ZnO-growth process is highly dependent on the complex combination of process parameters, e.g., system geometry (tube diameter) [21], temperature (*T*) [14], gas composition [15], and flow [15,22], pressure [21], type of substrate [23] and catalyst [12], the morphology expressions depend on the growth-setup. To differentiate between effects concerning the ZnO growth induced by the process parameters and those from laser irradiation of the catalytic gold thin film, ZnO-nanostructures were generated on unirradiated Au thin films with different layer heights (0.6–5.4 nm). Details about the thermal chemical vapor deposition (t-CVD) process are explained in detail in the Section 2. Figure 2a shows ZnO-nanostructures generated in a pure Ar–atmosphere (*p* = 12 mbar) at different temperatures *T* (hence different distances to the Zn-source, growth time *t* = 10 min). ZnO growth in pure Ar-atmosphere results in ZnO-nanorods (NRs) for all examined temperatures and gold layer heights. Analogue SEM micrographs for *T* = 1000 °C (upstream) are illustrated in the SI (Figure S3). The width and length of the NRs are dependent on the growth temperature. Lower temperatures lead to thicker, but shorter, NRs. Upon introducing 1% O_2_ to the carrier gas, a mixture of different morphologies consisting of thin nanowires (NWs) and narrow nanobelts (NBs) are obtained at *T* = 800 °C (Figure 2b). The colors in Figure 2 match the temperatures and sample positions illustrated in Figure 1.

**Figure 3:**
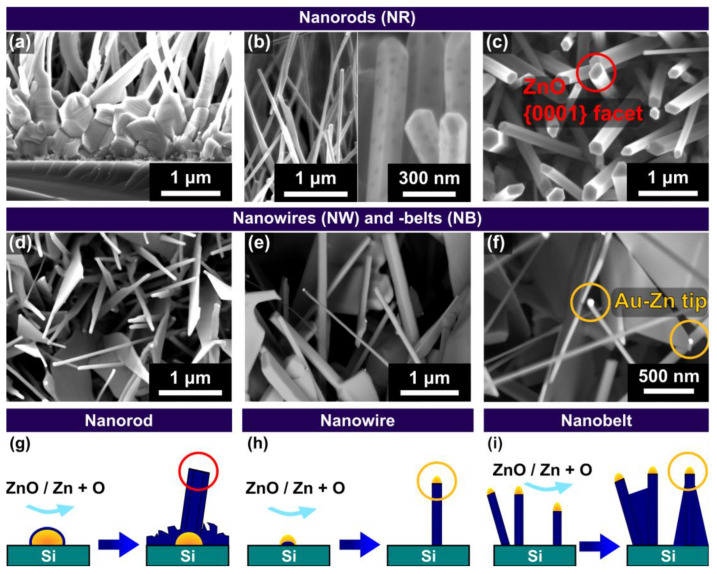
SEM micrographs (**a**,**b**: cross-sections) of ZnO-nanorods (NRs, **a**–**c**), -nanowires (NWs, **d**–**f**) and -nanobelts (NBs, **d**–**f**). High magnification images of NRs (**c**) show the flat ZnO {0001}-facets of VS-grown nanostructures, whereas the different VLS growth mechanism for NWs and NBs (**f**) is seen by the bright Au-Zn alloy particle at their tip. (**g**–**i**) Schematic representation of the growth mechanisms of NRs (**g**), NWs (**h**), and NBs (**i**). (dark blue: ZnO, cyan: Zn vapor, orange: Au, turquoise: Si-substrate).

The vapor-liquid-solid (VLS) growth process is characterized by gold nanoparticles [12] forming liquid Au-Zn catalyst droplets, resulting in tip growth of nanostructures from precipitated ZnO [11]. For the liquid-solid (VS) mechanism, the Zn directly reacts to ZnO at the surface, resulting in ZnO bases (root growth). Although the presence of a noble metal catalyst is not inherently necessary, the metallic nanoparticles act as energetically favored nucleation sites for the Zn-vapor [24]. Both growth mechanisms are explained in detail in the SI (Section S2). In Figure 3 the three main types of ZnO-nanostructure morphologies grown in our setup, i.e., nanorods (NRs, 1D), nanowires (NWs, 1D) and nanobelts (NBs, 2D), are exemplarily displayed. ZnO-NRs (Figure 3a–c) grow from a ZnO base layer formed by the VS mechanism, as schematically illustrated in Figure 3g. As a consequence, the NRs tip consists of {0001}-facets (Figure 3c), typical for the preferential c-plane growth ([0001]-direction) of the strongly anisotropic wurtzite crystal structure of ZnO [11]. NWs (Figure 3d–f) grow by the VLS mechanism, thus a gold particle is observed at their tip, as highlighted in Figure 3f and schematically represented in Figure 3h. NBs (Figure 3d–f) represent a two-dimensional ZnO growth, induced by excess Zn atoms, not dissolved in gold nanoparticles, leading to a growth of the sides of NWs. This growth is often initiated at the junction of two NWs as illustrated in Figure 3i [14].

We want to emphasize that although we designated the term NRs to VS-grown nanostructures and NWs to VLS-grown nanostructures to match the occurring morphologies for our growth conditions, in general, both mechanisms can lead to very thin (wire-like) and broad, faceted (rod-like) nanostructures. Comparing the morphology occurrence shown in Figure 2 with the growth mechanism assignments illustrated in Figure 3, it is obvious that a pure Ar-atmosphere leads to complete dominance of the VS mechanism, while the addition of O_2_ leads to a concurrent VS and VLs ZnO growth. This finding needs to be considered while investigating the growth of ZnO nanostructures on laser-generated catalysts. For further nanostructure characterization, Figure S4 (SI) shows an exemplary EDX measurement of ZnO nanostructures. All experiments compromising laser irradiation shown in the following part of the work were conducted on samples with a gold film thickness of 2.8 nm, representing a medium value of the explored thin film thickness range (Figure 2).

### 3.2. Au Catalyst Generation by Laser Irradiation

ZnO nanostructures grown on unirradiated samples, as discussed in the previous Section 3.1, display a shortcoming of the common (t-CVD) induced semiconductor growth. The substrate is homogeneously covered with similar noble metal catalysts, thus not allowing the growth of different ZnO nanostructure types on a single substrate. In literature, the spatial-selective generation of semiconductor growth is demonstrated, but again only by binary growth control [19,20,25,26]. To address this limitation we used pulsed laser-induced dewetting (PLiD) of the gold thin films before the thermally induced dewetting and ripening occurring in the ZnO growth process. Dewetting fragmentizes existing nanoparticles or -structures and ripening leads to the growth of one particle at the expense of others (by material transfer), all affecting the Au catalyst sizes. The scan strategy for areal-irradiation of the Au@Si-systems consists of a meandering scan path as explained in detail alongside the laser setup in the SI (Figure S2). 

**Figure 4:**
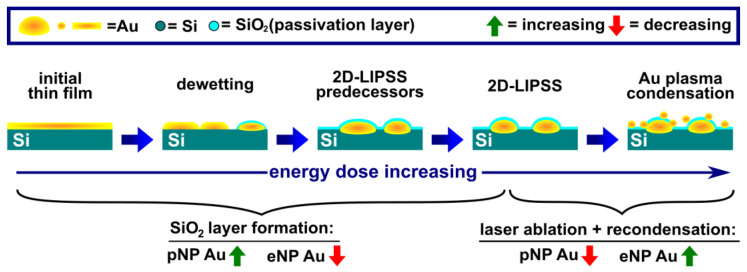
Schematic representation of the most important effects responsible for Au catalyst generation upon nanosecond laser irradiation. Displayed in order of their occurrence upon increased energy dose. Note that several effects may occur at the same time. Au nanoparticles (yellow) covered with a SiO_2_-layer (cyan) are designated to be passivated hence called passivated nanoparticles (pNPs), in contrast to exposed nanoparticles (eNPs) as known from traditional thermal dewetting.

Upon laser irradiation, multiple effects generating or modifying gold nanoparticles occur. At the lowest laser energies, the initial thin film layer is dewetted by (PLiD). Striving for the lowest surface energy, the film fragmentizes into smaller gold patches and nanoparticles. This matter reorganization is mediated through thin film and filament instabilities and results in the formation of increasingly smaller Au nanoparticles. To ensure the nanoparticle formation to be as homogenous as possible, we conducted all experiments using circularly polarized laser pulses. As a consequence, the formation of one-dimensional laser-induced periodic surface structures (1D-LIPSS) is suppressed. Nevertheless, 2D-LIPSS consisting of periodic arrays of relatively large, periodically arranged Au nanoparticles are formed at higher energy doses [27]. 2D-LIPSS nanoparticles are fully covered with a thin SiO_2_ layer and partly sunken in the silicon. Before the formation of the highly arranged 2D-LIPSS, their predecessor structures are formed. These Au nanostructures are not periodically arranged but are covered with a thin SiO_2_ passivation layer and are partly sunken into the silicon wafer too. At high laser energy doses, laser ablation of Au leads to the formation of a plasma. During the thermal relaxation following laser irradiation, the condensation of the aforementioned Au plasma leads to the formation of small nanoparticles. Unlike most other gold particles in this laser energy regime, these nanoparticles are not passivated by a SiO_2_ layer and are located on the sample’s surface. For all mentioned effects, the Au catalyst generation is schematically represented in Figure 4. PLiD, 2D-LIPSS generation, and the formation of small Au nanoparticles by condensation are extensively described in Durbach et al. [27]. The referenced work additionally illustrates the embedment of the various nanoparticles types in the substrate. Further explanations on the formation and properties of the passivating SiO_2_ layer are presented in Section 3.4. 

As the passivation of the Au nanostructures has significant implications on the ZnO growth, we classified the laser-generated nanoparticles into two categories. The first category consists of passivated nanoparticles, covered with a thin SiO_2_ layer and partly sunken in the Si-wafer, e.g., 2D-LIPSS and their predecessor structures. They are further referred to as pNPs (passivated nanoparticles). The second category of nanoparticles consists of exposed nanoparticles (eNPs) and includes particles generated by PLiD, as well as particles formed by Au plasma condensation. The sum of all presented laser-induced effects results in an initially increasing proportion of passivated Au. Due to laser ablation and recondensation of Au nanoparticles, this trend is reversed for the highest laser energy doses, e.g., effective pulse number *N*, as illustrated in Figure 4.

**Figure 5:**
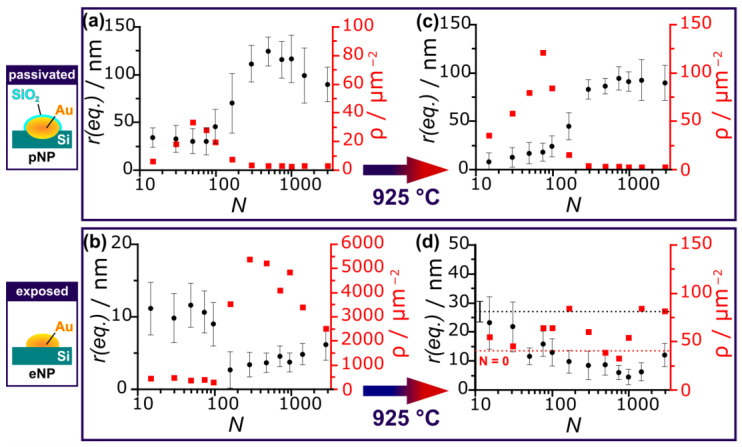
Equivalent radius *r(eq.)* (black) and area particle density *ρ* (red) of gold nanoparticles after laser irradiation (**a**,**b**) and after a subsequent heating process at a substrate temperature *T* = 925 °C (**c**,**d**) for both, passivated (pNPs, **a**,**c**) and exposed (eNPs, **b**,**d**) gold nanoparticles. The dashed lines represent the value for Au particles generated by the heating induced dewetting on unirradiated samples (*N* = 0).

The size and area density of both particle types were extracted from SEM images obtained by different detectors, as described in detail in the SI (Figure S5). To ensure comparability for uneven Au nanostructure shapes, the sizes are indicated by an equivalent to the radius of perfectly round particles possessing the same area. In Figure 5 the equivalent radius *r(eq.)* (black) and the area particle density *ρ* (particle count per area, red) are shown for particles generated by areal-irradiation using different effective pulse numbers *N*, a line-to-line distance (hatch) of *h* = 3.3 µm, a pulse fluence of $\varphi_{p}$ = 127 mJ/cm^2^ and a pulse frequency of *f* = 100 kHz. As ZnO-growth takes place at high temperatures, we examined the nanoparticle’s ripening and dewetting mediated by the temperatures present during the heating phase of the ZnO-growth process. A blank growth cycle, analog to the regular ZnO growth process, but using only trace amounts of ZnO/C, was conducted. As such the process conditions relevant to dewetting and ripening of the nanoparticles were controlled. The ZnO nanostructure growth was consequently inhibited, while still allowing preliminary ZnO-nucleation and Au-Zn alloying. This method enables a detailed study of the active catalyst particles and the temperature-mediated changes to *r(eq.)* and the area density *ρ* are observed by comparing Figure 5a/b to Figure 5c/d. The dashed lines in Figure 5d represent the values concerning Au particles generated only by heat-induced dewetting (unirradiated: *N* = 0).

Passivated nanoparticles (pNPs, Figure 5a) show a particle density peaking at about *N* = 50 ($\varphi_{p}$ = 127 mJ/cm^2^). For higher *N* the particle density converges to a value of about 3 particles/μm^2^ as a result of the self-regulated 2D-LIPSS formation. Upon increasing *N* the size of 2D-LIPSS nanoparticles increases to a maximum of ~124 nm (*N* = 498) before slightly diminishing, as expected for LIPSS generation [28]. This decrease in the pNPs’ size is explained by the partly ablation of the gold layer upon high energy doses, leading to the formation of small re-condensed Au-NP. At temperatures of 925 °C the pNPs undergo thermal dewetting, resulting in an increased count of smaller pNPs. Particles generated in the 2D-LIPSS generation parameter range, e.g., greater than *N* = 100 for $\varphi_{p}$ = 127 mJ/cm^2^, are relatively unaffected by the heating process, as they are well separated by the prior self-organization process. Exposed nanoparticles (eNPs, Figure 5b) show fairly constant particle sizes and densities for *N* smaller than 100, with an increase in the particle density by an order of magnitude and initial size drop for effective pulse numbers *N* greater than 100. This binary particle formation results from the aforementioned Au condensation from ns-laser laser ablation-generated plasma. Upon increasing *N*, the particle sizes (*r(eq.)*) decline constantly starting from a value of 27.1 nm for unirradiated samples. For the highest pulse numbers, a slight increase of *r(eq.)* is observed. After heating, the areal density *ρ(eNPs)* initially increases upon increasing *N* due to a dewetting process. After this initial increase, a dip in the particle density is observed. This laser parameter regime is optimal for 2D-LIPSS formation, resulting in an overall maximal proportion of Au residing in pNPs. Additionally, *ρ(eNPs)* the high particle density allows for additional particle fusing/ripening. 

In conclusion, progressively more Au is allocated to pNPs upon increasing *N,* leading to an increase in their size. As pNPs generated with higher *N* are already in a favorable energetic state, they are relatively unaffected by the heating process. The interplay of the dewetting of the initial thin film, emerging nanoparticles from laser ablation and thermal ripening, results in the density of eNPs fluctuating in a range of 40–80 particles/μm^2^, while their size shrinks. Only for the highest energy doses, a slight increase of *r(eq., eNPs)* is observed. Overall the size and area density of pNPs and eNPs are consistent with expectations concerning all laser irradiation effects summarized in Figure 4 and the subsequent thermal treatment.

**Figure 6:**
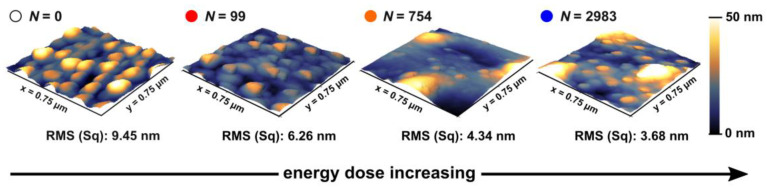
AFM measurements of gold-coated Si-wafer, irradiated with different effective pulse numbers *N* ($\varphi_{p}$ = 127 mJ/cm^2^) and subsequently heating to *T* = 925 °C. The RMS (Sq.) value indicates the surface roughness. For *N* = 2983 and *N* = 745 the roughness calculation was performed in the interstitial space between 2D-LIPSS. The color code for different pulse numbers corresponds to all images from Section 3.2 and Section 3.3, i.e. Figure 6, Figure 7, Figure 8 and Figure 9.

**Figure 7:**
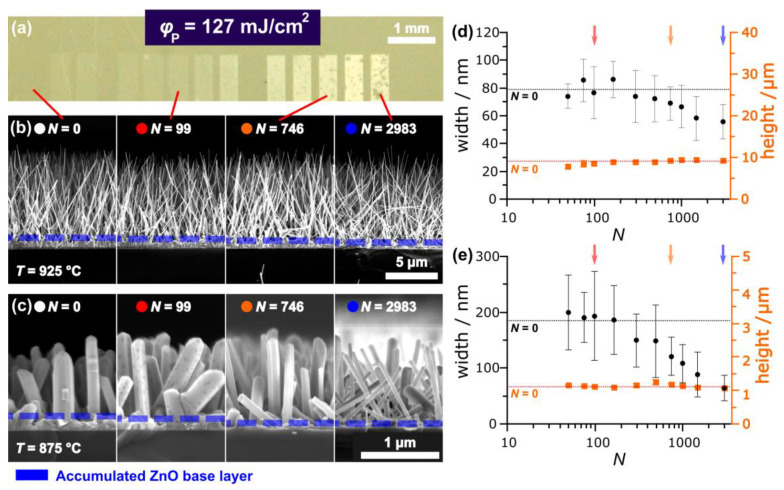
(**a**) Photography of a ZnO-NR covered Si-wafer compromising irradiated areas ($\varphi_{p}$ = 127 mJ/cm^2^, *t* = 10 min, *T* = 925 °C). (**b**) Scanning electron microscopy cross-sections of the sample shown in (**a**). (**c**) Cross-sections of shorter, wider ZnO-NRs ($\varphi_{p}$ = 127 mJ/cm^2^, *t* = 10 min, *T* = 875 °C) showing the same trend of thinning upon increasing *N*. (**d**,**e**) Width (black) and height (orange) of ZnO-nanorods shown in (**b**) respectively (**c**). The dashed lines in (**d**,**e**) represent ZnO-NR grown on unirradiated areas (*N* = 0).

**Figure 8:**
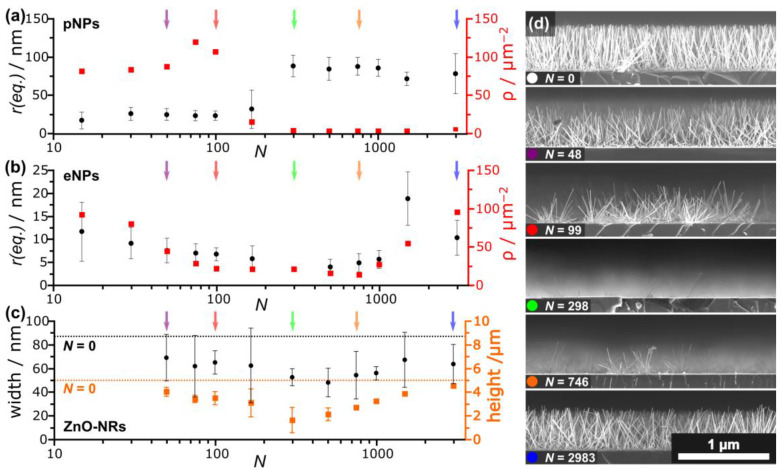
(**a**,**b**) Equivalent radius *r(eq.)* (black) and area particle density *ρ* (red) of gold nanoparticles ((**a**) pNPs, (**b**) eNPs) after laser irradiation and thermal treatment (925 °C). (**c**) Width (black) and height (orange) of ZnO-NRs. (**d**) Electron microscopic images (cross-sections) of ZnO-NRs generated with different effective pulse numbers *N*. ($\varphi_{p}$ = 160 mJ/cm^2^, *t* = 10 min, *T* = 925 °C).

**Figure 9:**
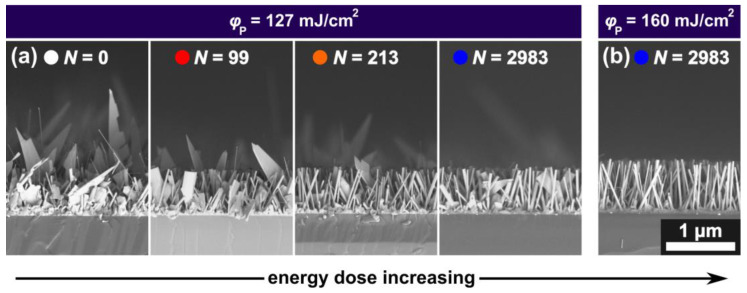
(**a**,**b**) SEM cross-sections of ZnO-nanostructures (Ar + 1% O_2,_ *t* = 10 min, *T* = 800 °C, (**a**) $\varphi_{p}$ = 127 mJ/cm^2^, (**b**) $\varphi_{p}$ = 160 mJ/cm^2^) showing a decrease of VLS-grown nanostructures (nanowires and -belts) upon increasing energy dose.

Atomic force microscopy (AFM) measurements were performed to acquire topographical images, as well as surface roughness information (RMS (sq.)), of the laser-modified catalysts for selected effective pulse numbers *N*. (Figure 6). The colored markers help to associate the data across several figures (Figure 6, Figure 7, Figure 8 and Figure 9). Note that for *N* = 745 and *N* = 2983 the roughness calculation was performed in the interstitial space between 2D-LIPSS to omit the relatively long-ranged surface modulation induced by the 2D-LIPSS formation (periodicity of ca. 510 nm), which is not assumed to have any notable effect on the ZnO nucleation. As for the reduction of the RMS values upon increasing *N*, less favorable nucleation for the Zn vapor on irradiated surfaces is expected. As a consequence, VS growth is inhibited.

### 3.3. ZnO Growth on Laser-Generated Catalysts

Concerning the root growth (VS) of ZnO nanostructures, Chandrasekaran et al. [29]. attributed the diameter of NRs/NWs to be dependent on the temperature *T*, the ratio of the semiconductor (e.g., Zn) at the interface, and the interfacial energy *σ*. The surface particle curvature [30], being a function of *r(eq.)*, and the surface roughness (RMS) [31] are both affected by laser irradiation, as investigated in Section 3.2. Consequently, all laser-irradiated catalysts show altered surface energies, i.e., nucleation properties, and are assumed to directly affect ZnO growth. Figure 7a,b shows an optical image and cross-sectional SEM images of ZnO-NRs respectively (Ar, *T* = 925 °C, *t* = 10 min). Figure 7c shows analogous cross-section micrographs of shorter, but wider ZnO-NRs grown at a lower temperature *T* = 875 °C. In Figure 7d/e the width (black) and height (orange) of the NRs from Figure 7b/c are shown. While the height (orange) remains relatively constant and similar to those grown on unirradiated samples (*N* = 0), a significant decrease in the NRs´ width (black) can be observed upon increasing *N*. Comparing the NRs´ width with the particle analysis data shown in Figure 5, the pNPs seem to have no significant influence on the VS-grown NRs. In contrast, the decrease in the NR’s-width (upon increasing *N*) correlates strongly with the diminishing of *r(eq., eNPs)*. Likewise, the error bars, narrowing for *r(eq., eNPs)*, correlate to more uniformly grown NRs (smaller error bars, Figure 7d,e). Although the VS mechanism responsible for the growth of NRs does not inherently require the presence of a gold catalyst, the exposed Au surface of the eNPs seems to be most influential for the ZnO growth. The Au surface is the energetically favored nucleation site [24]. An additional growth of a thick ZnO accumulated base layer (marked with blue dashed lines in Figure 7b,c and magnified in Figure 3a) is observed. This layer´s height diminishes continuously upon increasing the effective pulse number *N*, finely being barely visible for *N* = 2983. Consequently, the pulsed laser-induced dewetting (PLiD) of the Au catalyst layer may not only lead to a gradual control of the NRs´ width but also to an improvement of the NRs´ homogeneity and reduction of ZnO accumulations at the wafer surface. The effect of the laser irradiation on the NRs´ width is most apparent for thicker NRs as depicted in Figure 7c,e. 

Figure 8a,b shows *r(eq.)* and *ρ* of laser-generated catalysts formed using a higher pulse fluence of $\varphi_{p}$ = 160 mJ/cm^2^ and after heating (*T* = 925 °C). In Figure 8c the width and height of the corresponding ZnO-NRs are depicted. Similar to the laser irradiation with lower pulse fluences ($\varphi_{p}$ = 127 mJ/cm^2^, Figure 7), no noticeable effect of the pNPs on the ZnO-NRs´ growth or properties is observed, while the NRs´ growth is strongly influenced by the size of the eNPs. As aforementioned, the initial decrease of *r(eq.)* and *ρ* for eNPs is explained by the PLiD, while the re-increase is mediated by the condensation of Au from an ablation-induced plasma upon high laser energy doses. The higher pulse fluence of $\varphi_{p}$ = 160 mJ/cm^2^ allows for a significant change in the ZnO-NR density, induced by the significant decline of *ρ(eNPs)* and thus resulting in a nearly complete suppression of ZnO growth for *N* = 298 (Figure 8d). The suppression of VS-mediated ZnO growth is assumed to be also a consequence of the SiO_2_ layer formation.

The ZnO-NR growth shown above is dominated by the root-growth mechanism (VS), i.e., mediated by the surface’s eligibility as a nucleation site (surface energy *σ*). In contrast, the VLS process is mediated by a gold catalyst, alloying Zn from the environment and forming a ZnO-NW at its bottom (tip growth). As a consequence, the temperatures and the partial gas pressures at the substrate need to be in the correct range to allow Zn supersaturation and Au-Zn alloy formation. As discussed in Section 3.1, VLS-mediated ZnO growth was achieved by changing the partial gas pressures upon adding 1% of O_2_ to the Ar carrier gas. To obtain the nanostructures shown in Figure 9, samples were prepared and irradiated analogously as those shown in Figure 7 and Figure 8 and placed in the furnace using growth parameters favoring the VLS-growth mechanism (Ar + 1% O_2_, *T* = 800 °C, *t* = 10 min). Upon increasing the laser energy dose, a steep decline in the occurrence of VLS-grown ZnO nanostructures (NWs, NBs) is observed in the cross-sectional SEM images shown in Figure 9. 

The VLS growth is suppressed by two distinct laser-induced mechanisms. First of all the passivated 2D-LIPSS and their predecessors (pNPs) are partly sunken in the silicon wafer [27] and therefore bound to the substrate, impeding the ability of the catalyst to lift-off away from the surface and consequently inhibiting tip growth (VLS). Although not covered by a SiO_2_-layer, Au nanoparticles generated by laser ablation and recondensation are slightly embedded in the underlying SiO_2_ layer, also inhibiting a lift-off. For thermally dewetted metals, this interaction of the metallic nanoparticles with the substrate is commonly assigned to the contact angle [13]. Furthermore, laser irradiation leads to the formation of small Au nanoparticles (Figure 5 and Figure 7). For VLS growth the precursor activity needed to fulfill the necessary chemical potential is hardly ever reached for nanoparticles with a diameter smaller than 10 nm [32]. This critical particle diameter matches the smallest laser-generated particles in this work. Appropriate to both assumed VLS impeding mechanisms, Figure 9 shows the decrease in VLS-grown structures, i.e., the occurrence of NWs and NBs, upon higher laser energy doses. Incidentally, this also leads to a decrease in the overall ZnO nanostructure height, as VLS-grown nanostructures overgrow ZnO-NR as seen in Figure 9 for *N* = 0.

### 3.4. Effects of the Laser-Induced SiO_2_ Layer

**Figure 10:**
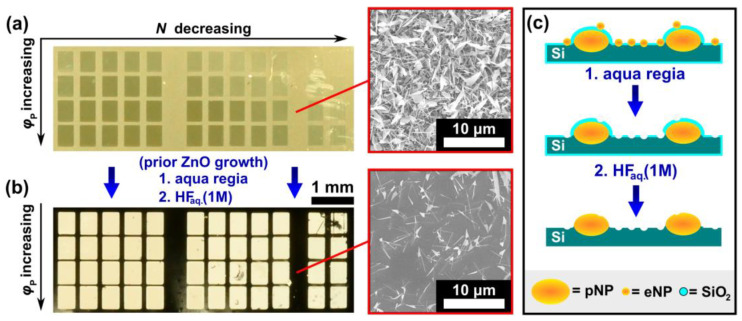
(**a**,**b**) Photography of an irradiated, initially gold-coated silicon wafer, overgrown with ZnO nanostructures. The wafer in (**a**) remained untreated after laser irradiation, whereas the wafer in (**b**) was consecutively treated with aqua regia and hydrofluoric acid before the ZnO growth. The visibly modified regions were irradiated with decreasing *N* (2984 to 15) from left to right and increasing pulse fluence $\varphi_{p}$ (112 to 160 mJ/cm^2^) from top to bottom (*t* = 10 min, *T* = 800 °C). (**c**) Schematic representation of the etching process including aqua regia and hydrofluoric acid, illustrating its effects on the gold nanoparticles and the silicon dioxide layer.

Additionally to the well-examined nanosecond (ns)-laser-induced changes for the Au catalysts, a change in the Si-surface through amorphization and oxidation, occurring during the 2D-LIPSS generation (ca. *N* > 100), may further influence the interfacial energy σ [27,28]. The laser-induced formation of a passivating SiO_2_ layer, well-known in ns-laser LIPSS formation literature [27,33,34], plays a significant role in the laser pulse-modified growth of ZnO nanostructures. A detailed description of the SiO_2_-layer and corresponding EDX analysis can be found in Reinhardt et al. [34]. The SiO_2_ layer existing on pNPs inhibits Au-Zn alloy formation and thus prevents the growth of VLS-induced ZnO-nanostructures. Simultaneously the roughness and surface energy are altered, consequently affecting the ZnO nucleation (VS). In Figure 10a a photograph of a Si-wafer, irradiated with different laser parameters, is displayed after ZnO-growth. Figure 10b shows an identically prepared Si-wafer, immersed in freshly prepared aqua regia (nitro-hydrochloric acid: 1:3 HNO_3_:HCl, 30 s) and subsequently immersed in hydrofluoric acid (HF, 1M, 60 s), before the ZnO growth process. The etching-process results in the removal of eNPs in the first and the removal of the SiO_2_ layer in the second step, laying bare the pNPs, e.g., 2D-LIPSS. The effects of both consecutive etching steps are schematically illustrated in Figure 10c. Contrarily to the untreated wafer seen in Figure 10a, the etched sample shows hardly any growth of ZnO nanostructures at unirradiated areas, as the gold thin film, acting as a catalyst, is completely removed. ZnO-nanostructures, seen as bright areas in Figure 10b, grow on the laid bare gold particles (formerly pNPs) situated at irradiated areas. Therefore ZnO growth is seen even for the lowest energy dose used in this work (top right corner of the substrate, $\varphi_{p}$ = 112 mJ/cm^2^, *N* = 15). The same etching technique allows for a spatially controlled growth of ZnO-nanostructures, as it removes the Au catalysts in all unirradiated areas, while the formerly passivated nanoparticles (pNPs) reside and are now exposed. This oppositional ZnO growth behavior can be further observed in a video of the growth process of untreated (left) and etched (right) samples at different temperatures (SI, Video S1). It should be noted, that the bonding of these exposed, formerly passivated nanoparticles to the substrate, only allow for VS-growth for the growth setup used in this work.

### 3.5. Overview of Laser-Irradiation’s Effects on ZnO Growth

**Figure 11:**
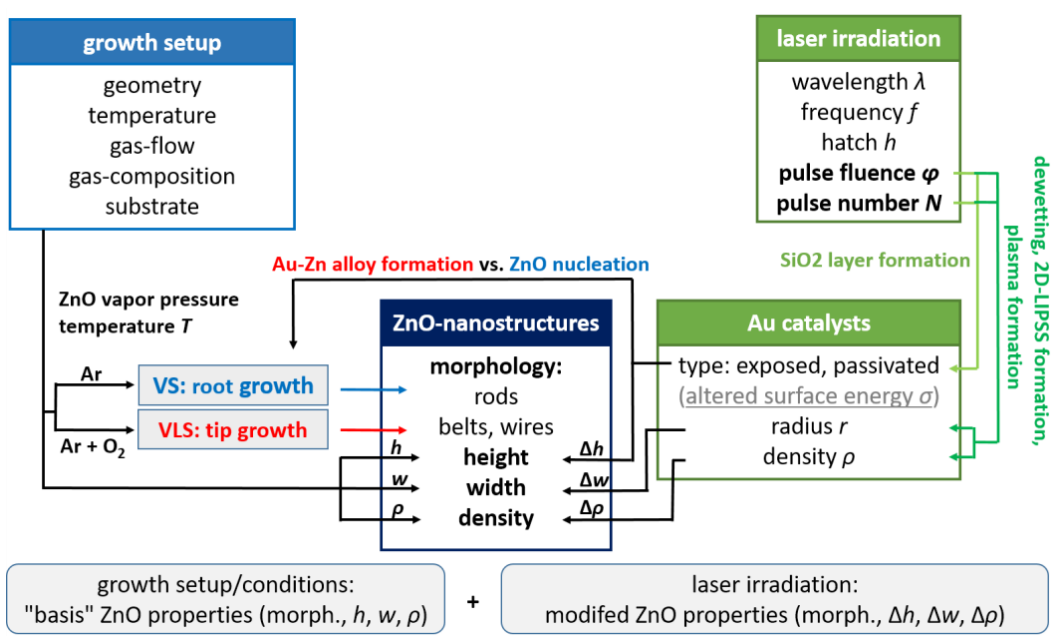
Schematic representation of the spatial-selective laser irradiation’s effect on ZnO nanostructure growth as compared to the growth setup and conditions. Note that additional cross-relations, which are of less importance and thus not illustrated, may occur.

As both, the laser-induced generation of gold catalysts and the subsequent ZnO growth compromise a plethora of different effects, the laser irradiation’s ability to affect ZnO growth should be recapitulated by highlighting the most important contributory factors. In general, the growth setup and the growth conditions determine the partial gas pressures of the reactive species in the vicinity of the substrate. Growth conditions are thus set for the entirety of the substrate, leading to predetermined ZnO nanostructure properties, e.g., morphology, height, width, and density. Laser irradiation of the Au surface before ZnO growth allows the generation of different Au catalysts. Depending on the laser parameters, e.g., pulse number *N* and pulse fluence $\varphi_{p}$, the size, density, and type of the laser-generated catalysts can be spatial-selectively controlled. For the system of Au@Si used in this work, two distinct types of Au-catalysts are generated. A part of the Au nanostructures is passivated with a thin SiO_2_ layer and/or partly embedded in the substrate. As a consequence, the surface energy *σ* and predisposition for Au-Zn alloy formation, and therefore the VS and VLs growth mechanisms, are altered locally (Figure 9). Likewise, the density and size of the catalyst (Figure 4 and Figure 5) have similar influences on the ZnO nanostructures’ width or density (Figure 7 and Figure 8). These parameters are a function of the applied laser energy dose. In Figure 11 the key aspects of the aforementioned interplay of ZnO growth conditions and the laser irradiation’s effects are schematically summarized.

**Figure 12:**
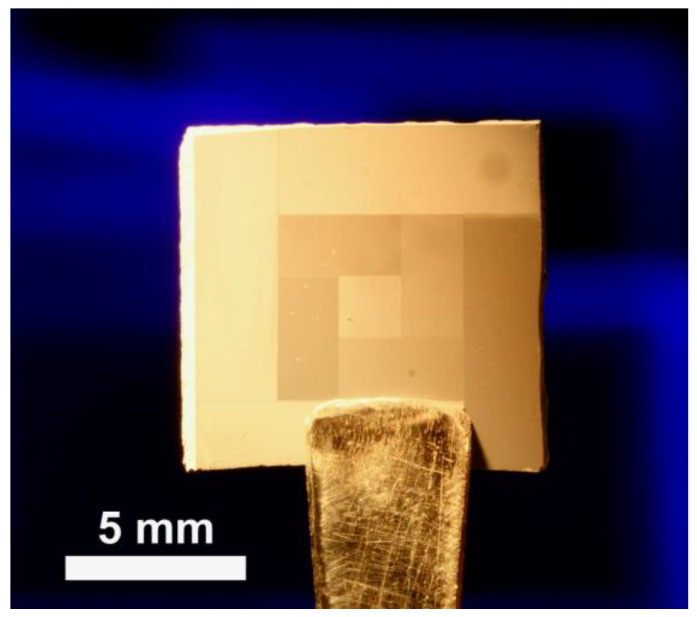
Photograph of a ZnO-nanostructure overgrown Si-wafer. The various shades correspond to different ZnO nanostructures grown on different laser-generated Au-catalysts.

The spatial-selective and gradual change of the irradiated areas is evident by observing the different shades, corresponding to macroscopic optical properties, shown for a ZnO-covered Si-wafer in Figure 12. Complementary, Figure S6 (SI) shows an overview of the boundary of an irradiated and unirradiated ZnO-overgrown area, illustrating the effect of the irradiation on the ZnO-NRs’ width and ZnO morphology-occurrence. The nanosecond laser-induced generation of Au catalysts shown in this work establishes a proof of principle for a maskless, controllable manipulation of ZnO nanostructures. By adjusting the irradiation conditions the presented principles are expected to be expandable to a variety of substrate materials. Likewise, a variation in the thickness of the underlying SiO_2_-layer could lead to additional growth control. By allowing better control of the growth mechanism, e.g., by changing to a pure Zn-source, the laser-induced changes to the VLS growth should become even more apparent. The control of ZnO properties achieved in this work may be explored for complex ZnO nanostructure designs for opto-electrical devices.

## 4. Conclusions

While the current state-of-the-art ZnO growth mostly relies on binary growth control, we successfully demonstrated ZnO structures grown on ns-laser-modified gold catalysts, resulting in a plethora of different ZnO-nanostructures on a single Si-substrate in a maskless process. The catalyst modification involves pulsed laser-induced dewetting (PLiD) and 2D-LIPSS (and predecessor structures) formation, leading to two distinct gold particle types. Passivated gold nanoparticles (pNPs) are partly sunken into the wafer and covered by a thin SiO_2_ layer leading to an active reduction in participating Au in the growth process. Exposed nanoparticles (eNPs) are playing a more active role in the ZnO growth process. Although their size and density ultimately change during the heating process through thermal dewetting and ripening, laser irradiation determines their final density and size. AFM measurements show additional modifications to the surface´s RMS value upon laser irradiation. Concerning ZnO nanostructures grown by a VS mechanism, we demonstrated the laser-induced alteration of the density and width of ZnO nanorods, as well as the height of an underlying accumulated ZnO layer. In the process-parameters regime favoring VLS growth, we demonstrated the laser-induced control of the morphology occurrence by gradually suppressing the growth of ZnO-nanowires and –belts. This spatial-selective control of ZnO nanostructures may allow the development of new optoelectronic nanodevices, with ZnO being a relevant but affordable semiconductor.

## Data Availability

I updated the data availability statement. The data that support the findings of this study are available from the corresponding author, N.H., upon reasonable request.

## Supplementary Materials

The following supporting information can be downloaded at: https://www.mdpi.com/article/10.3390/nano13071258/s1, Supplementary information on the experimental setup (Figures S1 and S2), ZnO growth and characterizations (Figures S3 and S4), the particle analysis (Figure S5), the influence of the laser irradiation on ZnO growth (Figure S6), and a video of the growth-process (Video S1) are available in the supporting information [11,12,14,15,21,22,23,24,27,35].
