# Supplementary material for: Laser-Induced Au Catalyst Generation for Tailored ZnO Nanostructure Growth
Source: Nanomaterials (Basel). 2023 Apr 2;13(7):1258. doi: 10.3390/nano13071258 (PMC10097237; doi:10.3390/nano13071258)
Supplement: Supplementary file 1 [file nanomaterials-13-01258-s001.zip › nanomaterials-2298451-supplementary.pdf]

Supporting Information

Laser-Induced Au Catalyst Generation for Tailored ZnO Nanostructure Growth

Sebastien Durbach [1], Lars Schniedermeyer [2], Anna Marx [3] and Norbert Hampp [4,*]

1. Experimental Setup

The heating rate of the furnace tube system shown in the main manuscript is shown in Figure 1a. This information is relevant as the ZnO growth may already start during the heating period of the system, influencing ZnO growth independently of the final substrate temperature.

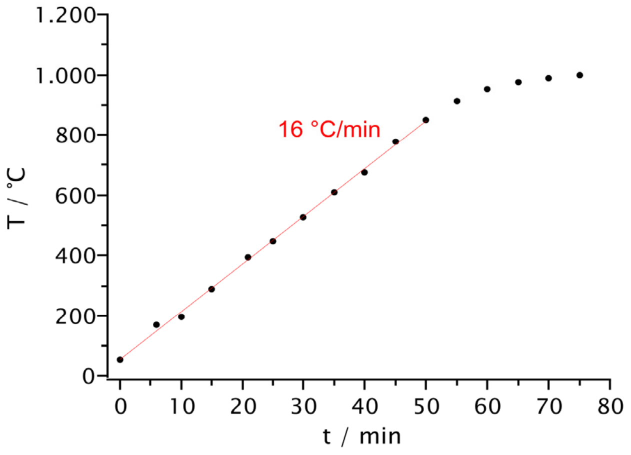

Figure S1: Temperature rise of the furnace system during the heating period (*t* = 60 min, ca. 16 °C/min).

In Figure S2 the experimental setup and scan strategy (inset) used for areal laser irradiation are shown. Power attenuation was achieved using the combination of a half-wave plate and a polarizing beam splitter. Circular polarization was obtained using a quarter-wave plate. The laser beam was scanned on the surface using a meandering scan path. The total energy applied was dependent on the laser pulse fluence φ_p (energy per area), the number of overlapping pulses in a single line N, the hatch h (line distance), and laser frequency f.

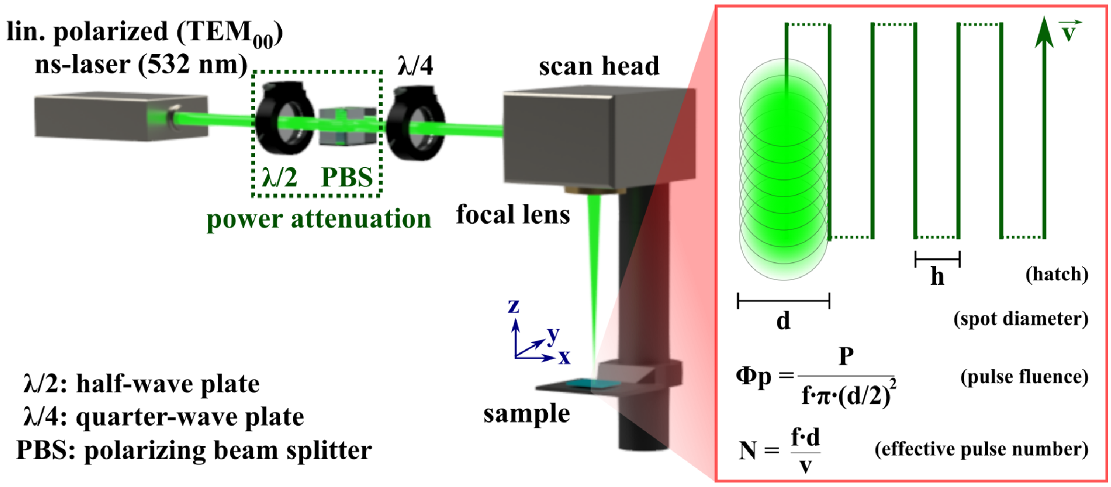

Figure S2: (Left) Schematic representation of the laser setup consisting of a half-wave plate and a beam polarizer for power control and a quarter-wave plate for polarization control. (Right) Scanning strategy used for irradiation of extended areas.

2. ZnO Growth

The ZnO nanostructures shown in this work were grown using the vapor–liquid–solid (VLS) or vapor–solid (VS) process. In the following section, additional information concerning both growth types is provided. The process names indicate the phase transitions the Zn species undergo during nanostructure formation. The VLS growth process is characterized by the presence of a noble metal particle [12], for example, Au, forming a liquid Au–Zn catalyst droplet by dissolving Zn in the initially pure metal catalyst. Upon supersaturation, Zn precipitates at the bottom of the alloy droplet, where it reacts in the presence of oxygen,. forming ZnO nanowires [11]. During this process, the catalyst droplet remains situated at the top of the nanowires (tip growth), further driving nanowire growth until the catalyst is fully depleted or the growth conditions are not met anymore. For the VS mechanism, the Zn directly reacts to ZnO at the surface, resulting in typically broad ZnO bases (root growth). For this process, the presence of a noble metal catalyst is not inherently necessary, although the gold nanoparticles act as energetically favored nucleation sites for the Zn vapor [24]. The formed ZnO prevents further VLS growth but may act as a nucleation site for several nanowires. The often found pseudo-hexagonal shape of thicker nanorods indicates that their main axis of preferential growth is orientated along the [0001]-direction (c-plane growth), as is typical for the strongly anisotropic wurtzite crystal structure of ZnO [11]. Generally, VS growth is slower (by 10 to 100 times) than VLS growth [11,21]. Both growth mechanisms often occur simultaneously, making the characterization of the ZnO nanostructure multifaceted and leading to discrepancies in the literature [14,21,35]. As the ZnO growth process is highly dependent on the complex combination of process parameters, e.g., system geometry (tube

diameter) [21], temperature (T)[14], gas composition [15] and flow [15,22], pressure [21], type of substrate [23], and catalyst [12], the individually obtained morphologies depend on the growth setup. Overall, the Zn vapor supersaturation and the oxygen concentration are accepted to be the critical factors determining whether the VLS or VS process dominates the nanostructure's growth [14,15,22].

Figure 2 in the main manuscript shows the growth of several ZnO nanostructures generated at different temperatures T and gas compositions. Figure S3 shows analog micrographs of ZnO nanostructures grown in an upstream position at 1000 °C.

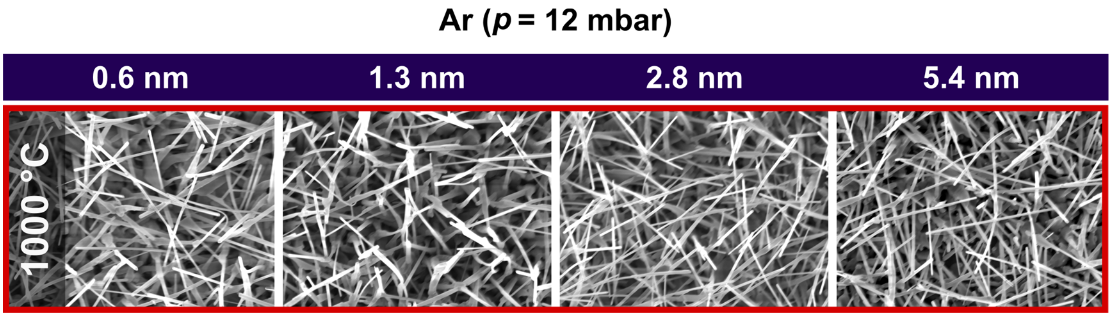

Figure S3: Typical ZnO nanostructures generated in a pure Ar atmosphere at T = 1000 °C (upstream) and gold thin-film layer heights (0.6–5.4 nm) (p = 12 mbar, t = 10 min, T_{source} = 1020 °C). The color matches the temperature and sample position illustrated in Figure 1 in the main manuscript.

Figure S4 shows an EDX elemental map of a cross-section of ZnO nanorods (NRs), with the centered ZnO accumulation layer at the bottom of the NRs. The blurring at the Si–ZnO interface was explained by a slight deviation of the measurement's angle from 90°. By comparing the elemental map of oxygen (green) and Zn (cyan), it was seen that the ZnO accumulation layer and the Zn NRs showed the same elemental composition, i.e., both consist of ZnO. The elemental map of gold shows the remainder of the gold nanoparticles at the nanostructure's root, indicating that the NRs were grown through the VS mechanism.

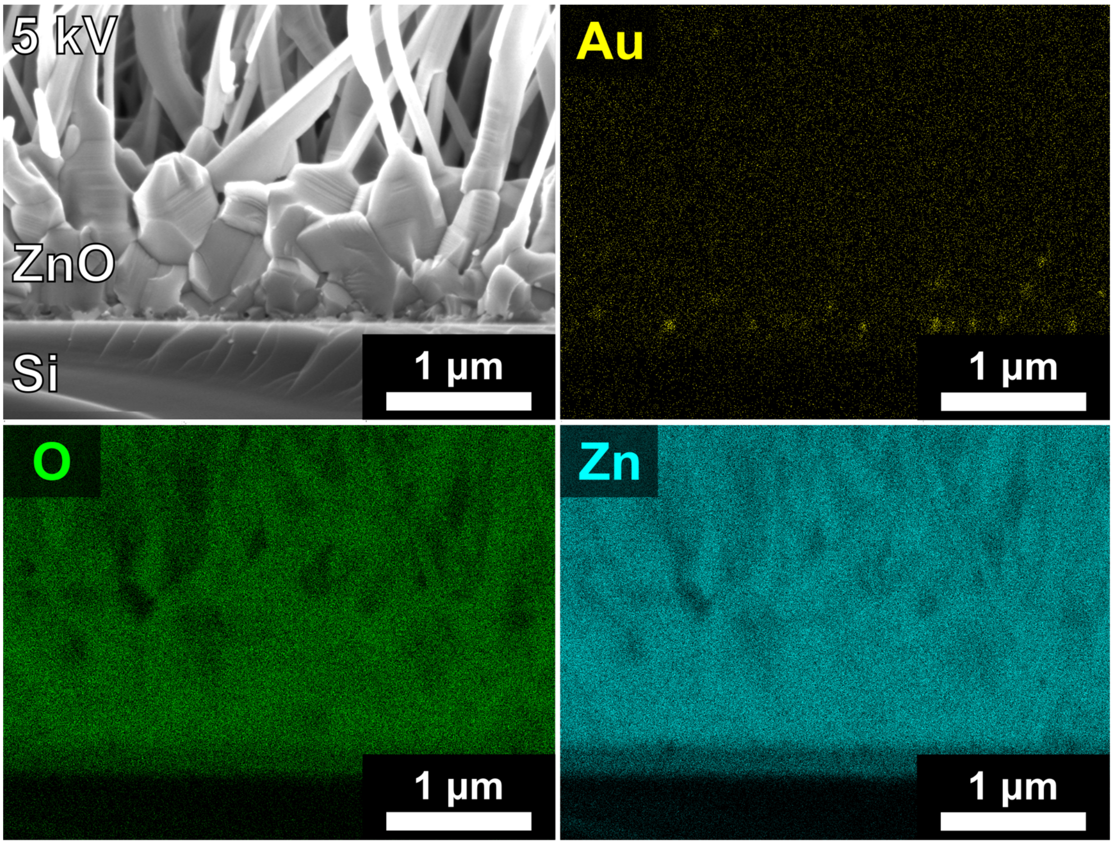

Figure S4: SEM image and corresponding elemental maps from EDX measurements of a ZnO accumulation layer and ZnO nanorods (yellow: Au; green: O; cyan: Zn). φ_p = 127 mJ/cm^2, N = 2984, p = 12 mbar (Ar), t = 10 min, T = 925 °C.

3. Particle Analysis

The particle equivalent radius *r(eq.)* represents the radius of a perfect circle with the same area as the particle. This allows us to account for uniform particle shapes. To extract *r(eq.).* from the two different particle categories, pNPs (passivated) and eNPs (exposed), masks representing the surface of the respective nanoparticles were created from SEM images. This process is shown in Figure S5a,b for pNPs and Figure S5c,d for eNPs. As the pNPs were covered with a thin SiO_2 layer and were partly sunken in the Si wafer, different contrasts were obtained in the SEM images using different detector systems. Figure S5e,f show SEM micrographs of the same particle-covered Si surface, taken with an "SE"(e) and "InLens"(f) detector. Both detectors detected secondary electrons, but were mounted at different positions. One detector was mounted in the beam line itself, while the other was at the side of the sample stage. While the eNPs did not show significant differences when comparing both images, pNPs showed a clear difference in contrast, appearing brighter in the SE detector. This difference allowed us to distinguish both particle types and extract the mask (g) for pNPs and the mask (h) for pNPs. Additional details of the pNPs' properties, mainly consisting of 2D-LIPSS and predecessors,

can be found in the work of Durbach et al. [27]. The validity of this method was confirmed using additional EDX measurements of both nanoparticle types.

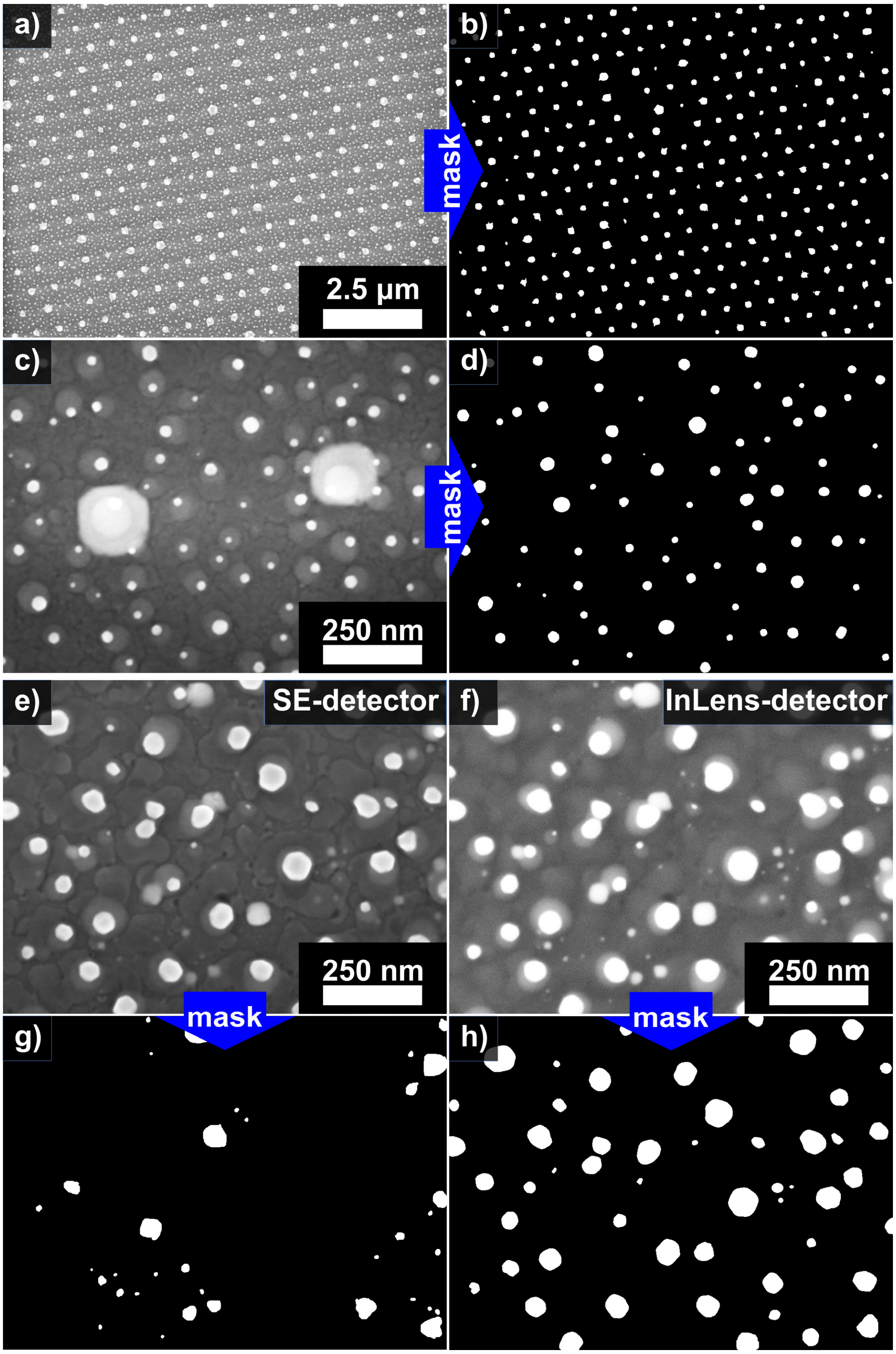

Figure S5: Examples of the particle analysis. (a,b) SEM image (a) and corresponding mask for pNPs (b). (c,d) SEM image (c) and corresponding mask for eNPs (d). (e,f,g,h) For more complex samples, the distinction between pNPs (g) and eNPs (h) could be made using the difference in the lateral SE (e) and Inlens (f) detector based on their shape and brightness.

4. Effects of Laser Irradiation on ZnO Growth

Figure S6 shows the edge between ZnO nanostructures grown at an unirradiated (top) and a laser-modified gold catalyst layer (bottom). The left image shows an example of a reduction in the ZnO NR width (φ_p = 127 mJ/cm^2, N = 2984, p = 12 mbar (Ar), t = 10 min, T = 925 °C). The right image shows an example of morphology suppression/selection through laser irradiation (φ_p = 127 mJ/cm^2, N = 2984, p = 12 mbar (Ar + 1%O_2), t = 10 min, T = 800 °C).

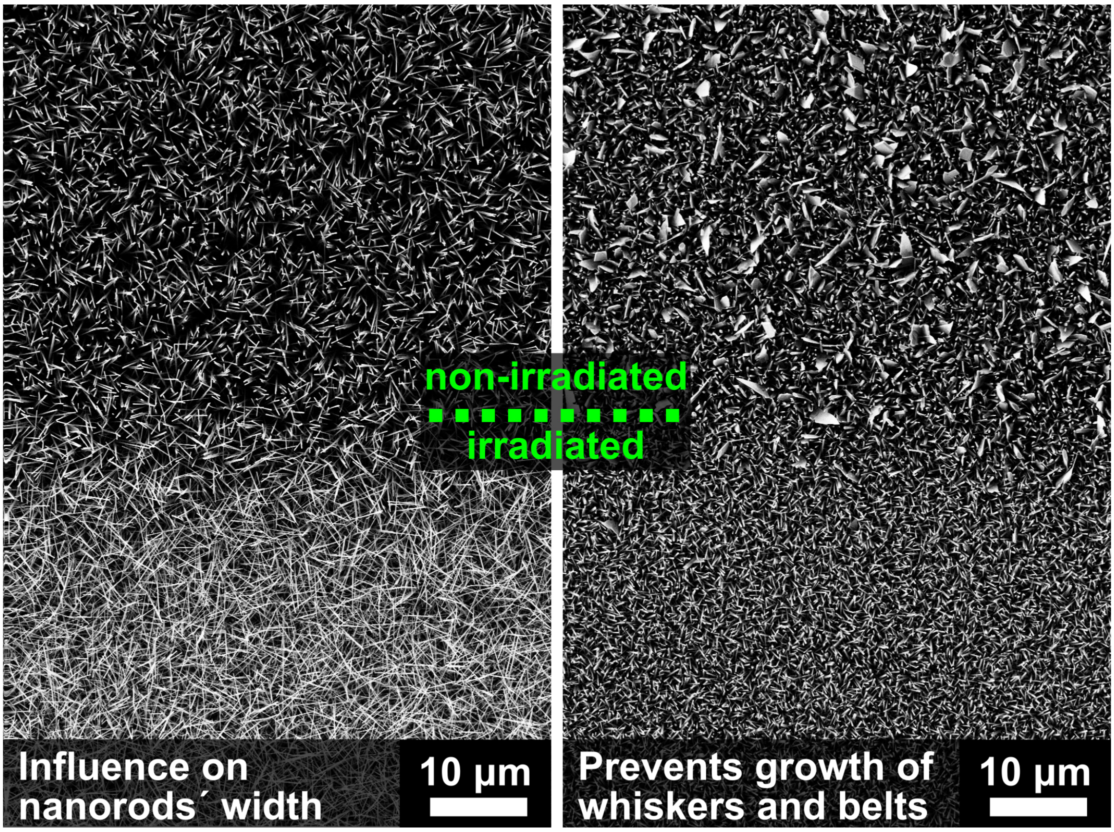

Figure S6: Border between an unirradiated (top) and irradiated (bottom) ZnO-overgrown area. (Left) Reduction of the ZnO NR width upon laser irradiation (φ_p = 127 mJ/cm^2, N = 2984, p = 12 mbar (Ar), t = 10 min, T = 925 °C). (Right) Reduction of the occurrence of ZnO NW and NB upon laser irradiation (φ_p = 127 mJ/cm^2, N = 2984, p = 12 mbar (Ar + 1%O_2), t = 10 min, T = 800 °C).

A clear difference in the growth process of irradiated and unirradiated areas at different temperature zones was observed macroscopically. We presented a time-lapse video (actual time is displayed at the bottom left) of the growth process on silicon wafers coated with a gold thin film and irradiated with different laser parameters. The video showed the growth starting before reaching the final process temperature as well as the growth rate to be dependent on the laser parameters (different rectangles). The wafers on the right were treated with hydrofluoric

acid (1M) and aqua regia, as described in the main manuscript (Figure 10). The time is displayed at the bottom left and the temperature at the bottom right of the video.

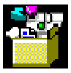

Video S1

References

11. Ramgir, N.S.; Subannajui, K.; Yang, Y.; Grimm, R.; Michiels, R.; Zacharias, M. Reactive VLS and the Reversible Switching between VS and VLS Growth Modes for ZnO Nanowire Growth. J. Phys. Chem. C 2010, 114, 10323–10329, doi:10.1021/jp909377b.
12. Zhang, Z.; Wang, S.J.; Yu, T.; Wu, T. Controlling the Growth Mechanism of ZnO Nanowires by Selecting Catalysts. *J. Phys. Chem. C* **2007**, *111*, 17500–17505, doi:10.1021/jp075296a.
14. Cheng, Q.; Ostrikov, K. Temperature-dependent growth mechanisms of low-dimensional ZnO nanostructures. *CrystEngComm* **2011**, *13*, 3455, doi:10.1039/c0ce00972e.
15. Burshtein, G.; Lumelsky, V.; Lifshitz, Y. The Role of Reactive Gases in ZnO Nanowires Growth via the Carbothermal Reaction. *J. Phys. Chem. C* **2016**, *120*, 15424–15435, doi:10.1021/acs.jpcc.6b03334.
21. Zhao, Y.; Li, C.; Chen, M.; Yu, X.; Chang, Y.; Chen, A.; Zhu, H.; Tang, Z. Growth of aligned ZnO nanowires via modified atmospheric pressure chemical vapor deposition. *Physics Letters A* **2016**, *380*, 3993–3997, doi:10.1016/j.physleta.2016.06.030.
22. Menzel, A.; Goldberg, R.; Burshtein, G.; Lumelsky, V.; Subannajui, K.; Zacharias, M.; Lifshitz, Y. Role of Carrier Gas Flow and Species Diffusion in Nanowire Growth from Thermal CVD. *J. Phys. Chem. C* **2012**, *116*, 5524–5530, doi:10.1021/jp212635w.
23. Murillo, G.C.; Bojorge, C.D.; Heredia, E.A.; Reca, N.W.d. Study of the Substrate Influence in ZnO Nanowires Oriented Growth. *Procedia Materials Science* **2015**, *8*, 630–634, doi:10.1016/j.mspro.2015.04.118.
24. Morris, R.J.H.; Dowsett, M.G.; Dalal, S.H.; Baptista, D.L.; Teo, K.B.K.; Milne, W.I. Spatial determination of gold catalyst residue used in the production of ZnO nanowires by SIMS depth profiling analysis. *Surf. Interface Anal.* **2007**, *39*, 898–901, doi:10.1002/sia.2610.
27. Durbach, S.; Hampp, N. Generation of 2D-arrays of anisotropically shaped nanoparticles by nanosecond laser-induced periodic surface patterning. *Applied Surface Science* **2021**, *556*, 149803, doi:10.1016/j.apsusc.2021.149803.
35. Nguyen, P.; Ng, H.T.; Yamada, T.; Smith, M.K.; Li, J.; Han, J.; Meyyappan, M. Direct Integration of Metal Oxide Nanowire in Vertical Field-Effect Transistor. *Nano Lett.* **2004**, *4*, 651–657, doi:10.1021/nl0498536.
